# Evolution of *Salmonella enterica* Virulence via Point Mutations in the Fimbrial Adhesin

**DOI:** 10.1371/journal.ppat.1002733

**Published:** 2012-06-07

**Authors:** Dagmara I. Kisiela, Sujay Chattopadhyay, Stephen J. Libby, Joyce E. Karlinsey, Ferric C. Fang, Veronika Tchesnokova, Jeremy J. Kramer, Viktoriya Beskhlebnaya, Mansour Samadpour, Krzysztof Grzymajlo, Maciej Ugorski, Emily W. Lankau, Roderick I. Mackie, Steven Clegg, Evgeni V. Sokurenko

**Affiliations:** 1 Department of Microbiology, University of Washington, Seattle, Washington, United States of America; 2 Institute for Environmental Health, Lake Forest Park, Washington, United States of America; 3 Department of Biochemistry, Pharmacology and Toxicology, Wroclaw University of Environmental and Life Sciences, Wroclaw, Poland; 4 Department of Animal Sciences, University of Illinois, Urbana, Illinois, United States of America; 5 Department of Microbiology, University of Iowa, Iowa City, Iowa, United States of America; Yale University, United States of America

## Abstract

Whereas the majority of pathogenic *Salmonella* serovars are capable of infecting many different animal species, typically producing a self-limited gastroenteritis, serovars with narrow host-specificity exhibit increased virulence and their infections frequently result in fatal systemic diseases. In our study, a genetic and functional analysis of the mannose-specific type 1 fimbrial adhesin FimH from a variety of serovars of *Salmonella enterica* revealed that specific mutant variants of FimH are common in host-adapted (systemically invasive) serovars. We have found that while the low-binding shear-dependent phenotype of the adhesin is preserved in broad host-range (usually systemically non-invasive) *Salmonella*, the majority of host-adapted serovars express FimH variants with one of two alternative phenotypes: a significantly increased binding to mannose (as in *S.* Typhi, *S.* Paratyphi C, *S.* Dublin and some isolates of *S.* Choleraesuis), or complete loss of the mannose-binding activity (as in *S.* Paratyphi B, *S.* Choleraesuis and *S.* Gallinarum). The functional diversification of FimH in host-adapted *Salmonella* results from recently acquired structural mutations. Many of the mutations are of a convergent nature indicative of strong positive selection. The high-binding phenotype of FimH that leads to increased bacterial adhesiveness to and invasiveness of epithelial cells and macrophages usually precedes acquisition of the non-binding phenotype. Collectively these observations suggest that activation or inactivation of mannose-specific adhesive properties in different systemically invasive serovars of *Salmonella* reflects their dynamic trajectories of adaptation to a life style in specific hosts. In conclusion, our study demonstrates that point mutations are the target of positive selection and, in addition to horizontal gene transfer and genome degradation events, can contribute to the differential pathoadaptive evolution of *Salmonella*.

## Author Summary

The process of *Salmonella* host-adaptation is traditionally considered to involve acquisition of novel genetic elements encoding specific virulence factors or loss of genes representing liability for the more pathogenic strains. Here, by analysis of the mannose-sensitive fimbrial adhesin FimH, we demonstrate that in addition to horizontal gene transfer and genome degradation, single amino acid replacement plays an important role in the differential adaptive evolution of *Salmonella*. We show that acquisition of specific structural mutations in FimH variants of host-adapted (systemically invasive) serovars results in either significantly enhanced or, alternatively, completely inactivated mannose-binding, whereas systemically non-invasive serovars retain a primordial relatively low-binding (shear-dependent) phenotype. A phylogenetic analysis indicates that these mutations are commonly of a convergent nature and occur under strong positive selection illustrating the role of point amino acid changes in convergent evolution of host-adapted *Salmonella*. Although we show increased bacterial adhesiveness and cell-invasiveness of the high-binding mutants, the physiologic role of the non-binding mutations in FimH remains to be determined.

## Introduction

*Salmonella enterica* is comprised of six subspecies (I, II, IIIa, IIIb, IV and VI) further subdivided into ∼2,500 serovars based on the presence of distinct surface antigens (somatic O, flagellar H and capsular Vi). The vast majority of *Salmonella* strains pathogenic to humans belong to subspecies I (*S. enterica* subsp. *enterica*), which is considered to be adapted to warm-blooded animals unlike the remaining subspecies which are found mostly in reptiles [1], [2]. However, among ∼1,500 serovars of subspecies I, relatively few cause severe systemically invasive infections while most serovars cause milder infections, usually limited to gastroenteritis. Heterogeneity in *Salmonella* virulence has been traditionally attributed to different distributions of various mobile genetic elements such as chromosomal pathogenicity islands, bacteriophages, transposons, plasmids, etc. [3], [4], [5]. However, the distribution of these factors does not correlate well with differences in clinical features. More recently, gene loss via deletion, insertional inactivation or truncation has been considered important in the evolution of highly pathogenic *Salmonella* [6], [7], [8]. In this study, however, we demonstrate that amino acid mutations in core genes of *Salmonella* are also a driving force behind pathoadaptive evolution of *Salmonella* serovars.

Various *Salmonella* serovars differ greatly in their host specificity and pathogenicity. Many serovars can infect a broad spectrum of animal hosts, typically producing self-limiting gastroenteritis (e.g., *S.* Enteritidis and *S.* Typhimurium). A small number of serovars exhibit narrow host-specificity and show increased virulence in their specific host that usually results in severe typhoid-like disease [9], [10]. For example, human adapted serovars Typhi, Paratyphi A and, Paratyphi C cause typhoid fever exclusively in humans and closely related primates. Avian adapted *S.* Gallinarum (biovars Gallinarum and Pullorum) cause severe systemic diseases in birds (fowl typhoid and pullorum disease respectively). Some other host-adapted serovars such as Choleraesuis or Dublin, although primarily associated with systemically invasive diseases in specific porcine and bovine hosts, respectively, rarely can infect other animal species including humans [11], [12], [13], [14]. It is noteworthy that in humans, infections with *S.* Choleraesuis and *S.* Dublin typically lead to severe invasive disease [14], [15], [16]. Similarly the D-tartrate negative biovar of Paratyphi B is primarily responsible for paratyphoid in humans but can be also sporadically isolated from dairy cattle [17], [18]. In contrast, the broad-host spectrum D-tartrate positive biovar of Paratyphi B (now biovar Java, formerly serovar Java) causes gastroenteritis in humans.

The evolution of pathogenic *Salmonella* from a non-pathogenic ancestor is primarily attributed to virulence genes acquired by horizontal gene transfer [3]. This includes the acquisition of large chromosomal regions (10–200 kbp) called *Salmonella* pathogenicity islands (SPI) that contain a number of functionally related genes [19]. Acquisition of small (<5 kbp) genetic loci, bacteriophages, and plasmids also contribute to the evolution of virulence [4], [5]. At least five *Salmonella* pathogenicity islands (SPI-1 to 5) have been identified in the serovars of *S. enterica* species, with a further nine islands with characteristics of SPIs identified in genomes of different serovars of subspecies I [20], [21]. Although the molecular effects of virulence traits encoded by these genetic loci have been studied in detail, their distribution does not simply correlate with host specificity or the level of systemic invasiveness of *Salmonella* serovars. Recently, genome sequencing studies of host-adapted serovars *S.* Typhi, *S.* Gallinarum, *S.* Choleraesuis and newly emerging systemically invasive strains of *S.* Typhimurium in sub-Saharan Africa revealed that these bacteria undergo extensive gene deletion and truncation [6], [7], [8], [22], [23]. Because the majority of lost genes have functional orthologs in systemically non-invasive *Salmonella* with key roles in intestinal colonization, it has been suggested that narrow host-adaptation of *Salmonella* co-evolved with the loss of an intestinal life style and acquisition of the ability to survive in a systemic niche [22].

While high-throughput comparative genomics of different *Salmonella* strains have identified a large number of single nucleotide polymorphisms (SNPs), very little is known about the functional consequence of these sequence variations and their potential impact on *Salmonella* ecology and pathogenicity [24], [25], [26]. SNPs that represent random spontaneous mutations in the coding or regulatory regions of genes can result in modification or loss of gene function and/or expression [27]. These so called ‘change of function/loss of function’ mechanisms can confer a strong selective advantage to bacteria during their spread and growth in diverse host environments, improving their survival or increasing their pathogenic potential and thus driving their evolution toward an enhanced pathogenic phenotype. Therefore they are referred to as pathoadaptive mutations [27]. A well characterized example of pathoadaptative mutation is allelic variation in the FimH adhesin of type 1 fimbriae expressed by uropathogenic *Escherichia coli* [28]. FimH mediates mannose-sensitive bacterial adhesion to target cells [29]. It has been demonstrated that uropathogenic isolates, due to structural point mutations in *fimH*, express variants of FimH with an increased capability to bind monomannose receptors, conferring a significant advantage for colonization of the bladder compared to most commensal *E. coli* [30], [31].

Like *E. coli*, *Salmonella* have mannose-specific type 1 fimbriae with a tip-associated adhesin also termed FimH [32], [33]. Despite functional and semantic similarity, the fimbriae in these two species are not evolutionarily related, with virtually no significant sequence homology, including *fimH* [34], [35]. The FimH adhesin is an excellent candidate for the study of evolutionary changes in *Salmonella* adaptation. This adhesive protein has been reported to play an important role in *Salmonella* adhesion and invasion [36], [37], [38], [39], [40], [41], [42], and recently was shown to be a crucial mediator of bacterial transcytosis through M-cells, a process which is of great relevance in triggering the mucosal immune response [43], [44]. The expression of FimH on the bacterial surface is tightly controlled by the *fim* regulatory proteins (FimZ, FimY and FimW) in response to environmental signals [45], [46], [47] and, due to involvement of *fim* regulatory proteins in the global regulatory network, FimH expression is coupled with the expression of other virulence factors including flagella [48], T3SS [49], [50], [51] and LPS [52]. Thus, it can be expected that diversity of host cell receptors, the host immune response and other indirect mechanisms exert differential selective pressures on FimH adhesins during different types of *Salmonella* infection.

Recent studies on FimH adhesins of *S.* Typhimurium demonstrated that there is allelic variability of FimH, where single amino acid substitutions increase bacterial binding to human HEp-2 and murine DC cells, and increase efficiency of biofilm formation in the small intestine of mice [38], [53]. Also, in *S.* Gallinarum (biovars Pullorum and Gallinarum), FimH variants have lost the ability to mediate mannose-sensitive adhesion due to a single point mutation that eliminates mannose binding [54]. However, the general pattern of FimH variability across different serovars of *Salmonella* is unknown, and it is not established whether point mutations in FimH are acquired under positive selection and thus are likely to contribute to the evolution of virulence in *Salmonella*, especially in strains capable of invasive infections.

To determine whether point mutations in FimH are associated with pathoadaptive evolution of specific *Salmonella* serovars, we performed genetic and functional analyses of FimH adhesin variants from 33 serovars of *S. enterica*. We found that FimH in host-adapted (systemically invasive) serovars evolve in a convergent way, both structurally and functionally, highlighting the role of point mutations in the differential adaptive evolution of *Salmonella*.

## Results

### The phylogenetic relatedness of *fimH* genes within *S. enterica*

**Figure 1:**
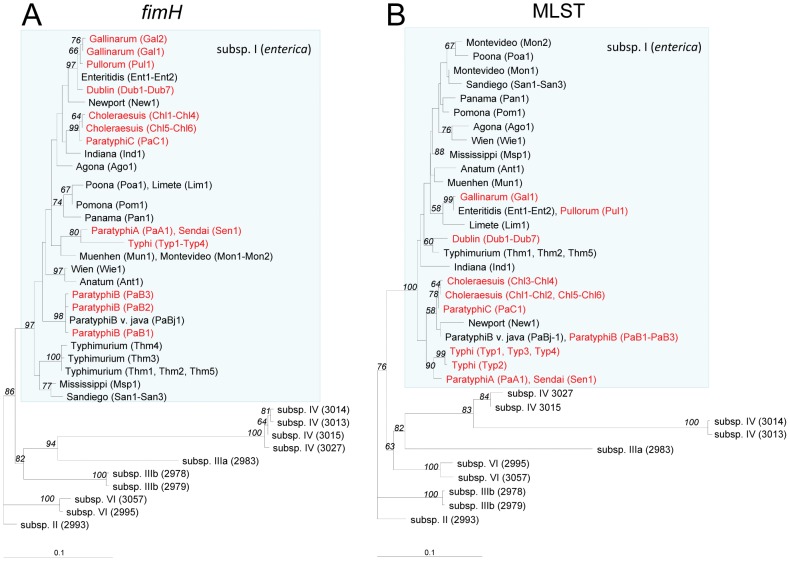
Maximum-likelihood DNA phylograms of *S. enterica fimH* and concatenated MLST loci (*aroC*, *hisD* and *thrA*). The *fimH* tree (A) was built based on an alignment of *fimH* sequences amplified from 55 isolates including 45 different strains of subspecies I and 10 strains of subspecies II–VI (for details see Table 1). Five additional alleles of *fimH* obtained from GenBank (*S.* Typhimurium AJB3 (Thm3), *S.* Typhimurium LB5010 (Thm4), *S.* Gallinarum 287/91 (Gal1) and 589/02 (Gal2), and *S.* Paratyphi C 49 [RKS4594] (PaC1) were included. The MLST loci tree (B) was built on an alignment of concatenated sequences of three genes (*aroC*, *hisD* and *thrA*) obtained for 57 study strains. The trees shown were rooted using *S. enterica* subsp. II (2993). The italicized values along the branches denote % bootstrap values based on 1000 runs (the bootstrap proportions along the terminal branches separating isolates within single serovars as well as the ones below 50% are not shown). Systemically invasive serovars are shown in red and non-invasive serovars are shown in black. Strain tags are as used in the text.

*fimH* was amplified from 55 of 56 *S. enterica* isolates, of which 45 represented 22 serovars of subspecies I, and 11 isolates represented other subspecies (Table 1). The isolate of *S. enterica* subsp. IIIa (2980) carried only part of the *fimH* gene and was excluded from further analysis. The maximum-likelihood phylogenetic tree constructed based on 55 amplified *fimH* sequences and five additional *fimH* alleles obtained from GenBank (*fimH* of *S.* Typhimurium AJB3 and LB5010, *S.* Gallinarum 287/91 and 589/02, and *S.* Paratyphi C 49 [RKS 4594]) is presented in Figure 1A. The *fimH* sequences of subspecies I (*enterica*) were grouped in a distinct phylogenetic clade separate from *fimH* of subspecies II–VI, which were also separated from one another (with bootstrap values for branch separation higher than 60%).

**Table 1:** List of bacterial strains used in this study.

Strain	Strain tag	Strain origin/characteristic relevant for this study (year of strain isolation)	Reference/source
***Wild-type:***			
*S.* Typhimurium SL1344	Thm1	Bovine	[96]
*S.* Typhimurium 1 (RKS4194)	Thm2	Human, England	[97]
*S. enterica* serogroup B C-24	Thm5	Human stool, recurrent gastroenteritis/typhoid fever, (2007) *	F. Fang^1^
*S.* Pomona DMSO63	Pom1	Unknown	[98]
*S.* Anatum DMSO13	Ant1	Unknown	[98]
*S.* Dublin DMSO30	Dub1	Unknown	[98]
*S.* Dublin C-29	Dub2	Human blood, colitis/sepsis, (2011)	F. Fang
*S.* Dublin 10351	Dub3	Bovine (necropsy) (2006)	M. Samadpour^2^
*S.* Dublin 11170	Dub4	Bovine spleen (2006)	M. Samadpour
*S.* Dublin 11087	Dub5	Bovine lung (2006)	M. Samadpour
*S.* Dublin 10928	Dub6	Bovine feces (2006)	M. Samadpour
*S.* Dublin 10828	Dub7	Bovine necropsy (2006)	M. Samadpour
*S.* Mississippi DMSO49	Msp1	Unknown	[98]
*S.* Agona DMSO09	Ago1	Unknown	[98]
*S.* Newport DMSO55	New1	Unknown	[98]
*S.* Pullorum SL297	Pul1	Unknown	[98]
*S.* Paratyphi B S66	PaB1	Unknown	[74]
*S.* Paratyphi B S957	PaB2	Unknown	[74]
*S.* Paratyphi B 2421	PaB3	Unknown	S. Clegg^3^
*S.* ParatyphiB var java DMSO45	PaB-j1	Unknown	[98]
*S.* Enteritidis 11122-1	Ent1	Avian	M. Samadpour
*S.* Enteritidis 12154-1	Ent2	Bovine (environmental)	M. Samadpour
*S.* Choleraesuis χ3246	Chl1	Porcine	[99]
*S.* Choleraesuis 10411-1	Chl2	Bovine lung (necropsy)	M. Samadpour
*S.* Choleraesuis 11175-1	Chl3	Porcine lung (necropsy)	M. Samadpour
*S.* Choleraesuis 11359-1	Chl4	Porcine (necropsy)	M. Samadpour
*S.* Choleraesuis 1656/04	Chl5	Porcine	M. Ugorski^4^
*S.* Choleraesuis 1500/05	Chl6	Porcine	M. Ugorski
*S.* Typhi 63 (RKS3333)	Typ1	Dakar	[100]
*S.* Typhi 64 (RKS3320)	Typ2	Dakar	[100]
*S.* Typhi C-23	Typ3	Human blood, typhoid fever (2002)	F. Fang
*S.* Typhi Ty2 (JSG624)	Typ4	Unknown	J. Gunn^5^
*S.* Paratyphi A 42 (RKS4993)	PaA1	Laboratory strain (ATCC 9150)	[100]
*S.* Sendai 58 (RKS2866)	Sen1	Human, California	[100]
*S.* Limete 47 (RKS3215)	Lim1	Human, Africa	[100]
*S.* Indiana 25 (RKS4250)	Ind1	Scotland	[100]
*S.* Muenchen 32 (RKS3121)	Mun1	Laboratory strain (ATCC 8388)	[100]
*S.* Montevideo 30 (RKS1762)	Mon1	Human, Georgia	[100]
*S.* Montevideo 31 (RKS 1740)	Mon2	Human, Florida	[100]
*S.* Wien 71 (RKS4000)	Wie1	Human, France	[100]
*S.* Poona MI14a	Poa1	Marine Iguana, S. Plaza, Galapagos	R.I. Mackie^6^
*S.* Panama LI03e	Pan1	Land Iguana, S. Plaza, Galapagos	R.I. Mackie
*S.* Sandiego MI08d	San1	Marine Iguana, S. Plaza, Galapagos	R.I. Mackie
*S.* Sandiego LI23b	San2	Land Iguana, Santa Fe, Galapagos	R.I. Mackie
*S.* Sandiego LI010a	San3	Land Iguana, S. Plaza, Galapagos	R.I. Mackie
*S. enterica* subsp. II (RKS 2993)	2993	Unknown	[97]
*S. enterica* subsp. IIIa (RKS 2980)	2980	Corn snake, Oregon	[97]
*S. enterica* subsp. IIIa (RKS 2983)	2983	Human, California	[97]
*S. enterica* subsp. IIIb (RKS 2978)	2978	Human, Oregon	[97]
*S. enterica* subsp. IIIb (RKS 2979)	2979	Human, California	[97]
*S. enterica* subsp. IV (RKS 3015)	3015	Animal, Canal zone	[97]
*S. enterica* subsp. IV (RKS 3027)	3027	Human, Illinois	[97]
*S. enterica* subsp. IV (RKS 3013)	3013	Tonga-T1	[97]
*S. enterica* subsp. IV (RKS 3014)	3014	Human, Florida	[97]
*S. enterica* subsp. VI (RKS 2995)	2995	India	[97]
*S. enterica* subsp. VI (RKS 3057)	3057	Unknown	[97]
***Recombinant:***			[97]
*S.* Typhimurium SL1344H3	-	*fimH*::kan mutant of *S.* Typhimurium SL1344	[38]
*S.* Typhimurium LBH4	-	*fimH*::kan mutant of *S.* Typhimurium LB5010	[101]
*S.* Typhi Ty57	Typ4	aroA::kan mutant of *S.* Typhi Ty2	This study

1 Harborview Medical Center, Seattle, WA, USA.

2 Institute for Environmental Health, Lake Forest Park, WA, USA.

3 University of Iowa, Iowa City, IA, USA.

4 Wroclaw University of Environmental and Life Sciences, Wroclaw, Poland.

5 Ohio State University, Columbus, OH, USA.

6 University of Illinois, Urbana, IL, USA.

*fimH* phylogeny was compared to the phylogenetic relationship of the study strains based on their Multi-Locus Sequence Typing (MLST) profiles by using concatenated sequences of three housekeeping genes *aroC*, *hisD* and *thrA* (Figure 1B). Strains belonging to same serovars had the same, usually distinct, MLST profiles and there was a general congruency between the *fimH* and MLST trees. In particular, different subspecies of *S. enetrica* were split into distinct clades on both *fimH* and MLST trees and, within subspecies I, the *fimH* phylogeny of serovar clades like Typhi and Paratyphi A, Choleraesuis and Paratyphi C, or Pullorum, Gallinarum, and Enteritidis corresponded well with MLST genotype. Also within the subspecies I, *fimH* of the same serovars were grouped together, suggesting limited horizontal transfer of *fimH* among different serovars. However, there was less congruency between the serovar clades defined by *fimH* and MLST, indicating that, in general, *fimH* of different host-adapted, systemically invasive serovars have evolved along independent, phylogenetically unlinked pathways.

### Structural variability of *S. enterica* subspecies I FimH

**Figure 2:**
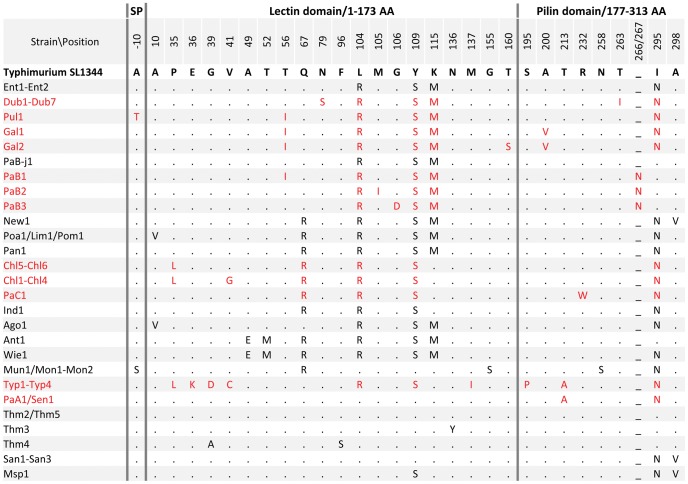
Amino acid variation in *S. enterica* subspecies I FimH. Residues identical to the amino acid sequence of *S.* Typhimurium SL1344 (Thm1) FimH are indicated by dots. Systemically invasive serovars are shown in red and non-invasive serovars are shown in black. Position -10 represents the position in the signal peptide of unprocessed FimH upstream of the cleavage site.

The average nucleotide diversity of *fimH* from *S. enterica* subspecies I was 1.7±0.2%, with 28 distinct alleles. The majority of the nucleotide polymorphisms were point mutations with a seven-fold higher rate of synonymous over nonsynonymous mutations. The only other polymorphism type was a three-nucleotide insertion (aat) between nucleotide position 864 and 865 in three *fimH* alleles of Paratyphi B (PaB1, PaB2 and PaB3). There were a total of 27 protein variants encoded by *fimH* with an overall protein level identity of 96.6% (Figure 2). There were 45 amino acid replacements in the 335 amino acid polypeptide and an asparagine insertion between positions 266 and 267 in Paratyphi B FimH variants. The majority of serovars contained unique FimH variants, but some serovars, e.g., Montevideo and Muenchen; Paratyphi A and Sendai; or Poona, Limete and Pomona, shared the same protein variants of FimH (Figure 2). For some serovars, e.g. Paratyphi B, Choleraesuis and Gallinarum, within-serovar variability of FimH was observed. FimH variations affected the putative leader peptide (22 aa long) as well as a predicted mannose-binding lectin domain (N-terminal 173 aa in the mature protein) and shaft-anchoring pilin domain (C-terminal 136 aa). There was no significant domain clustering or predominance of either conservative or nonconservative protein changes among FimH variants overall or between FimH from systemically invasive and non-invasive serovars (Figure 2).

**Figure 3:**
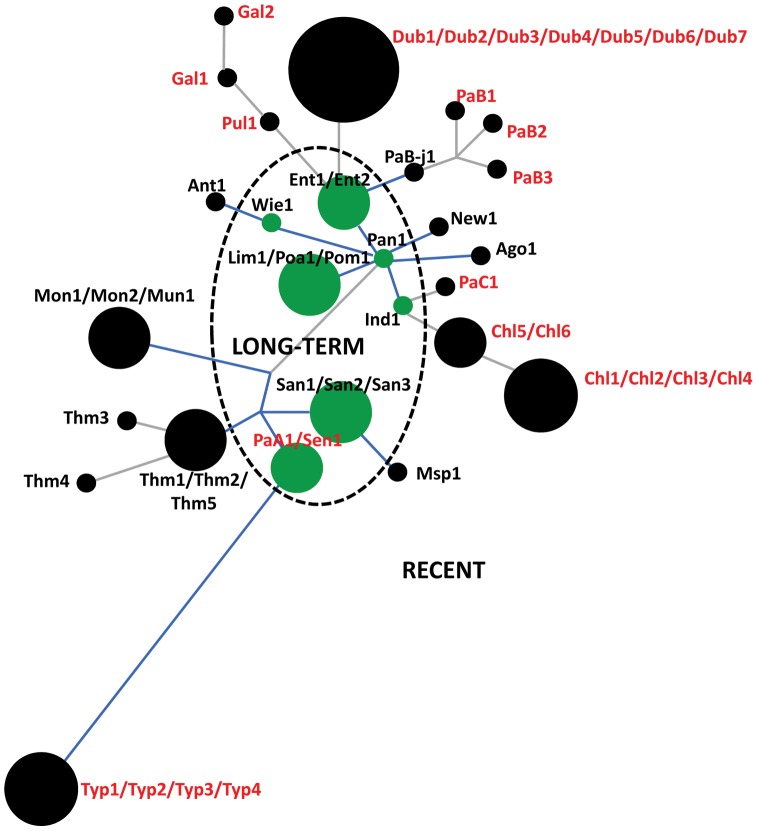
DNA-based protein phylogram of *S. enterica* FimH, derived from ZP analysis. The tree was built based on the 50 *fimH* sequences of *S. enterica* subsp. I. Each circle represents a unique structural variant, and the size of the circle is proportional to the number of representative sequences. The dashed line separates the long-term (green) from the recently emerged variants (black). Branches marked in blue indicate branches containing synonymous mutations. The length of each branch is proportional to the number of non-synonymous mutations that were acquired. The strain tags of systemically invasive serovars are in red and the non-invasive serovars in black.

When the FimH protein variants were analyzed for emergence from an evolutionary perspective, most FimH variants (20 out of 27) appeared to have emerged relatively recently, without accumulation of silent changes in the coding alleles (Figure 3). The rest of the FimH variants were of a relatively long-term evolutionary origin, with accumulation of silent changes in the corresponding alleles or along the surrounding branches on the tree. Interestingly, while the systemically non-invasive serovars included nine recent and six long-term FimH variants, only one of twelve FimH variants from systemically invasive serovars was of long-term origin (p = 0.04). Moreover, alleles of the majority (10 of 12) of FimH variants from systemically invasive serovars evolved from the nearest allele exclusively by structural mutations.

Thus, while the overall pattern of distribution of FimH variations was not strikingly different between systemically invasive and non-invasive serovars, the evolutionarily recent origin and structural nature of the *fimH* mutations appeared to be much more typical for the systemically invasive serovars.

### High-adhesive and inactive variants of FimH are selected in systemically invasive serovars of *S. enterica*

**Figure 4:**
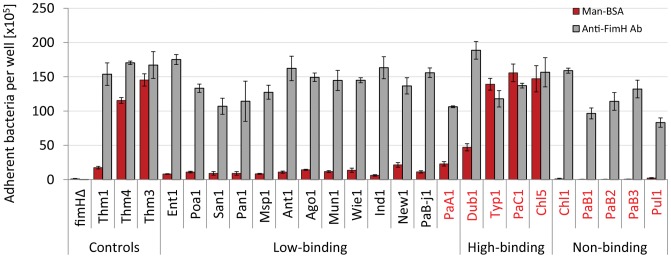
Binding phenotypes of natural *S. enterica* subspecies I FimH variants. Static adhesion of *S.* Typhimurium LBH4 transformed with plasmids encoding different variants of FimH or plasmids carrying *fimH* deletion (fimHΔ) to Man-BSA (red bars) and anti-FimH^SE^ antibody (grey bars). The binding of ^3^H- labeled bacteria was determined as described in Material and Methods. Data are the means ± SD of triplicates from one representative experiment of three experiments that were performed. The strain tags of systemically invasive serovars are in red and the non-invasive ones in black. Bacterial binding was >95% inhibitable in the presence of 50 mM methyl-D-mannopyranoside (not shown). * The non-binding FimH variants of *S.* Gallinarum were not tested in this study.

We next compared functional properties of different structural variants of FimH by examining the level of bacterial binding to Mannose-BSA (Man-BSA) in an isogenic system. Man-BSA, used as model substrate, contains single (mono-) mannose residues, Man1, covalently coupled to BSA. As controls for assessing the level of binding, we used a *fimH*-knockout variant (fimHΔ) as well as three structural variants of *S.* Typhimurium FimH characterized previously: a relatively low-binding variant of *S.* Typhimurium SL1344 (Thm1) and two high-binding variants from strains AJB3 (Thm3) and 5010 (Thm4) [38], [53], [55] (Figure 4). The majority of FimH variants exhibited relatively low but specific (>95% mannose-inhibitable) binding to Man-BSA (Figure 4 and data not shown). However, with the exception of FimH from *S.* Paratyphi A/Sendai, all of the low binding FimH variants came from systemically non-invasive serovars of *S. enterica*. In contrast, all high-binding FimH variants were from systemically invasive serovars such as Typhi (strains Typ1–Typ4), Paratyphi C (PaC1), and Choleraesuis (strains Chl5–Chl6). Also, FimH from another systemically invasive serovar Dublin (Dub1–Dub7) bound Man-BSA significantly stronger than low-adhesive FimH variants, though the binding was weaker in comparison to the other high-binding FimH variants. The binding differences were not due to a difference in the expression level of FimH, as bacteria expressing different variants of FimH bound relatively well to polyclonal anti-FimH antibody (Figure 4). Interestingly, FimH variants expressed by the remainder of the invasive strains did not exhibit detectable mannose-specific binding to Man-BSA, and also failed to bind Man5 oligosaccharides carried by ribonuclease B (RNase B, Figure S1), to which all functionally-active FimH variants bind with much higher affinity than Man1 ligands [55]. However, they still retained their interaction with anti-FimH antibodies (Figure 4). Such an ‘inactive’ FimH phenotype may be similar to that shown previously for FimH variants of *S.* Gallinarum biovars Pullorum (Figure 4 and Figure S1) and Gallinarum ([54], not tested in this study).

We also performed the static adhesion assay to examine mannose-dependent binding of wild type strains of *Salmonella*. As presented in supplementary Figures S2 A and B, the mannose-binding pattern of wild type strains corresponded to the binding pattern of FimH variants expressed in isogenic recombinant system. The wild-type *Salmonella* carrying *fimH* alleles encoding low-binding FimH exhibited relatively weak binding to monomannose substrate (Man1) and bacteria with ‘high-binding’ *fimH* alleles adhered strongly to monomannose (Figure S2 B, red bars). Wild-type isolates with non-binding *fimH* alleles did not adhere to any of the mannosylated substrates tested (Man1 and Man5). The differences in the level of mannose-specific adhesion between these three groups of wild-type *Salmonella* were clear, even though variability in the fimbriation level was observed (Figure S2, grey bars). A reduced level of fimbriation was found particularly for isolates of serovar Choleraesuis (Figure S2) while isolates of Partyphi A and Sendai from this study did not produce fimbriae at all (data not shown). Nevertheless, the fimbriated wild-type and recombinant strains displayed the same receptor specificity of binding as assessed by determination of the Man1/Man5 binding ratio (Figure S3).

Selected FimH variants with a range of binding activities (Typ1, PaC1, San1, Chl1 and Pul1) were further analyzed in Man-BSA binding under flow conditions (Figure S4). FimH variants with low-binding phenotypes mediated shear-enhanced (shear-dependent) adhesion to Man-BSA, whereas FimH variants with high-binding phenotypes bound to Man-BSA in a shear-independent manner. Bacteria expressing non-binding variants of FimH did not exhibit binding to Man-BSA under any flow conditions. These results indicate that the low-binding shear-activated phenotype of FimH is predominant among systemically non-invasive serovars of *S. enterica*, while high-adhesive shear-independent or inactive FimH variants occur only in systemically invasive serovars of *Salmonella*.

### FimH of systemically invasive *Salmonella* evolved by accumulation of “activating” and/or “inactivating” point mutations

**Figure 5:**
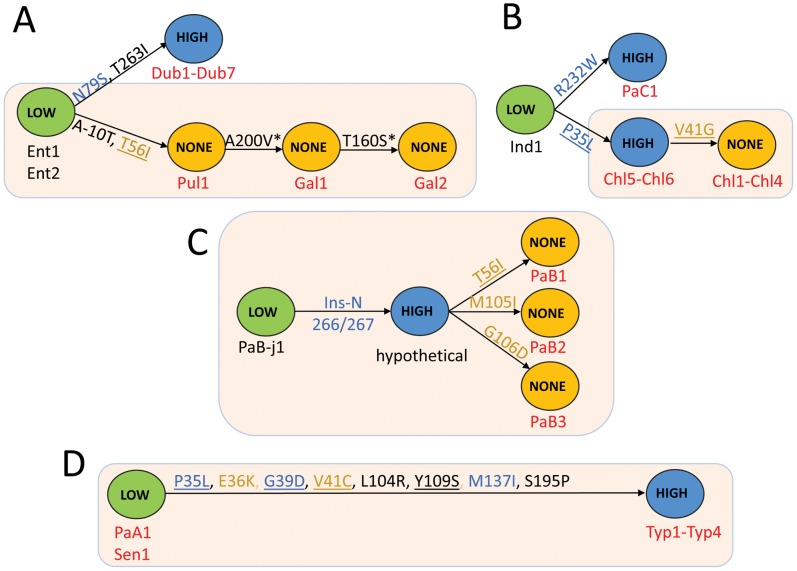
Schematic representation of evolutionary changes in the FimH binding phenotype of selected *S. enterica* serovars. The evolutionary changes in the FimH of *S.* Enteritidis, *S.* Pullorum, *S.* Gallinarum and *S.* Dublin (A), *S.* Indiana, *S.* Paratyphi C and *S.* Choleraesuis (B), *S.* Paratyphi B (C) and *S.* Paratyphi A, *S.* Sendai and *S.* Typhi (D). Low (green)-FimH with low-binding phenotype; High (blue)-FimH with high-binding phenotype; None (orange)-inactive variant of FimH. The strain tags of systemically non-invasive serovars are in black and the systemically invasive serovars in red. Structural mutations are given along each arrow. Structural hot-spot mutations are underlined. The activating mutations are in blue and the inactivating mutations are in orange. The FimH variants from strains with phylogenetic relatedness supported by MLST are shown in tan boxes.

We compared amino acid sequences of high-binding and inactive FimH variants with their closest FimH ancestors exhibiting low-binding phenotypes (Figure 1). According to the *fimH* phylogeny, the low-binding phenotype is evolutionarily ancestral to the high-binding FimH and, in most cases, non-binding variants evolved from high-binding variants (Figure 5). For example, high-binding variants of FimH of *S.* Paratyphi C and *S.* Choleraesuis (Chl5–Chl6) evolved from the low-binding variant of *S.* Indiana; and the non-binding FimH of *S.* Choleraesuis (Chl1–Chl4) evolved from the high-binding FimH of *S.* Choleraesuis (Chl5–Chl6). In the case of *S.* Paratyphi B, the whole spectrum of phenotypes evolved within the serovar. The non-binding phenotype of *S.* Gallinarum biovar Pullorum appeared to evolve from low-binding variants of *S.* Enteriditis, with the former then giving rise to two non-binding FimH variants of *S.* Gallinarum biovar Gallinarum. Interestingly, with the exception of *S.* Typhi FimH, all microevolutionary changes in the gene observed in systemically invasive serovars occurred exclusively via amino acid replacements, i.e., without accumulation of any silent mutations, suggesting the action of strong positive selection. In addition, some of the mutations occurred repeatedly (P35L, T56I) or in the same amino acid position (V41G and V41C) indicating convergent evolution of the FimH variants, also a strong indicator of positive selection. Importantly, the evolutionary changes of the *fimH* variants correspond to serovar phylogeny based on MLST data (Figure 5, light orange boxes).

The naturally occurring mutations were separately introduced into a plasmid-encoded *S.* Typhimurium SL1344 (Thm1) *fimH* (low-binding phenotype) and assayed for functional effects in the isogenic *S.* Typhimurium LBH4 strain. In the isogenic system, a switch from low- to high-binding phenotype was observed for the single mutations N79S (Ent1/Ent2 to Dub1–Dub72), P35L and R232W (Ind1 to Chl5/Chl6 and to PaC1, respectively) and the N266/267 insertion (PaB-j1 to hypothetical PaB variant) (Figure S5 and Figure 5). Mutation T56I resulted in a change from low- (Ent1/Ent2) to non-binding phenotype (Pul1), while a high- to non-binding switch was confirmed for T56I, M105I and G106D (all in *S.* Paratyphi B) as well as V41G in *S.* Choleraesuis. Notably, the non-binding mutation V41G had a deleterious effect on bacterial fimbriation when introduced directly into the low-binding FimH test background (*S.* Typhimurium SL1344). However, when this mutation was introduced into the high-binding P35L background of its immediate ancestor, fimbriation was normal as indicated by anti-FimH antibody binding while the binding function was lost (Figure S5). When the multiple mutations leading from the low-binding phenotype of *S.* Paratyphi A/Sendai FimH to the high-binding FimH of *S.* Typhi were tested individually, three mutations resulted separately in a high-binding phenotype: P35L (as in Chl5–Chl6), G39D and M137I (Figure S5). In contrast, mutations E36K and V41C resulted individually in a significantly decreased or a non-binding phenotype, respectively. Unlike V41G in *S.* Choleraesuis, the V41C substitution did not eliminate fimbriation in the FimH test background.

Taken together, these results indicate that both high- and non-binding phenotypes of FimH in systemically invasive serovars of *S. enterica* are acquired under positive selection by convergent evolution, with inactivation of FimH generally preceded by evolution of the high-binding phenotype.

### FimH variations affect adhesion to and internalization into epithelial cells and macrophages

**Figure 6:**
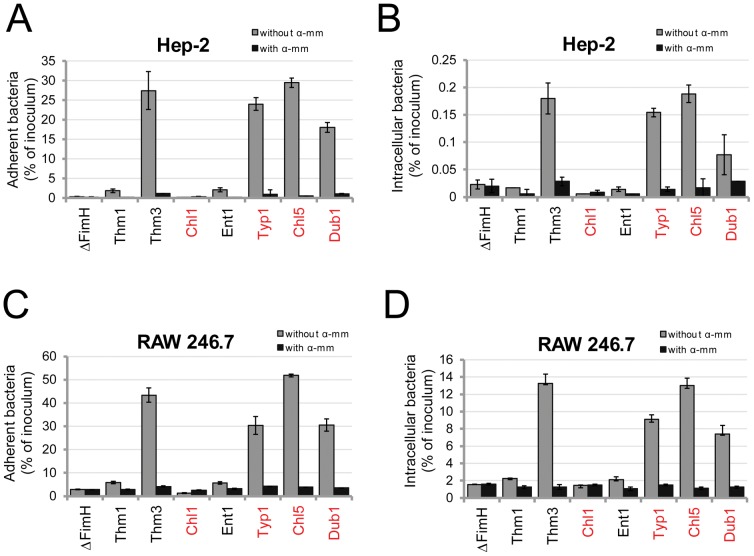
FimH-mediated bacterial interaction with epithelial and macrophage cell lines. Bacterial adhesion to (A and C) and invasion of (B and D) Hep-2 cells (A and B) and RAW264.7 cells (C and D). Different variants of FimH were expressed in *S.* Typhimurium SL1344H3 and bacterial binding was tested in the absence and presence of 50 mM methyl-D-mannopyranoside (α-mm). Data are the means ± SD of triplicates from one representative experiment of three experiments that were performed.

We investigated how variation in mannose-binding by *Salmonella* FimH affects bacterial cell adhesion and invasion. The human epithelial cell line HEp-2 and the murine macrophage cell line RAW264.7 were used as target cells. We compared isogenic strains that express two previously characterized *S.* Typhimurium FimH variants, low-binding FimH^SL1344^ (Thm1) and high-binding FimH^AJB3^ (Thm3), that differ in a single amino acid N136Y, and FimH variants from five other serovars: Enteritidis (Ent1, low-binding), Dublin (Dub1, medium-binding), Choleraesuis strain Chl5 and Typhi strain Typ1 (both high-binding), and Choleraesuis strain Chl1 (non-binding). Bacterial adhesion and invasion were assessed after allowing a bacterial suspension to interact with the target cell monolayers under static conditions without centrifugation. As shown in Figure 6, the level of bacterial binding to both epithelial cells (6A) and macrophages (6C) corresponded well to the mannose-binding capability of the FimH variants, with the high-binding FimH mediating up to 10-fold higher adhesion than the low-binding variants and up to 100-fold higher adhesion than inactive FimH of Choleraesuis (Chl1) or the FimH knockout strain that does not express type 1 fimbriae (fimHΔ). The binding was strongly inhibited by a soluble mannose derivative (methyl-alpha-D-mannopyranoside). Thus, under our experimental conditions cell adhesion is primarily mediated by FimH, and the variants with activating mutations mediate significantly better cell binding. When bacterial internalization was assessed (Figure 6B and D), the pattern generally was the same. Consequently, the highly-adhering bacteria were internalized to a significantly higher degree than the low-adhering bacteria. However, while the level of FimH-dependent bacterial adhesion to these two types of eukaryotic cells was similar, the invasion level differed significantly with 75 times greater invasion of the macrophage cell line (7.4–13.2% of bacterial inoculum) compared to the epithelial cells (0.07–0.19% of bacterial inoculum). Again, invasion was strongly inhibited by soluble mannose.

Although *in vitro* differences in cell adhesion and invasion were observed for bacteria expressing different FimH variants, no differences in viable bacterial counts from spleen and liver were observed 7 days after oral inoculation of BALB/c mice with *Salmonella* strains expressing FimH variants (data not shown).

## Discussion

In the present study, we have demonstrated that the FimH adhesin in highly pathogenic (systemically invasive) *S. enterica* serovars undergoes convergent evolution via point mutations that are likely to have adaptive significance for *Salmonella* ecology and pathogenesis. We have shown that the shear-dependent low-binding phenotype of FimH is preserved in serovars typically associated with gastroenteritis, whereas the majority of systemically invasive serovars carry FimH variants that exhibit one of two alternative but evolutionarily-interconnected phenotypes: increased affinity towards mannose or the outright loss of the mannose-binding activity. The functional diversification of FimH in systemically invasive *Salmonella* results from repeated, independently acquired structural mutations showing that, in addition to horizontal gene transfer and gene deletion, point mutations are the target of strong positive selective pressure and contribute to the pathoadaptive evolution of *Salmonella*.

While separation of *Salmonella* into systemically invasive, host-adapted and systemically non-invasive, broad host-range serovars may seem somewhat arbitrary, this distinction is generally consistent with prevailing views on *Salmonella* ecology and epidemiology [9], [10]. Subspecies II–VI of *Salmonella* (*salamae*, *arizonae*, *diarizonae*, *houtenae*, and *indica*) that are almost exclusively isolated in nature from reptiles only sporadically cause infections in humans [1], [2], [56]. Subspecies I (*enterica*) is much more pathogenic for humans than the other five subspecies and is typically isolated from warm-blooded animals. However, the ecology of subspecies I serovars is not as distinct as originally thought, as many (e.g. Poona, Pomona, Abaetetuba, Newport), commonly inhabit wild reptiles, often as dominant *Salmonella* serovars [57]. Some of the subspecies I strains tested here (serovars Poona, Panama and Sandiego) were isolated from free-living marine or land iguanas from the Galapagos Islands. Thus, many and probably most of the subspecies I serovars truly have a broad range of natural hosts, not limited to warm-blooded animals. However, other serovars such as Typhi, Paratyphi A–C and Choleraesuis are much more distinct in their host association and ability to cause systemic (invasive) disease relative to other serovars of subspecies I. Interestingly, the distinct structural and functional characteristics of FimH in the serovars defined here as systemically invasive support the physiologic validity of their grouping.

To identify adaptive changes in *S. enterica* FimH, we first used a bioinformatics-based approach called Zonal Phylogeny that is highly sensitive in detecting footprints of positive selection, superior in this regard to more conservative tests such as the dN/dS ratio [28], [58]. Zonal Phylogeny detects an excess of recently evolved protein variants and determines whether or not they evolved via hot-spot mutations (repeated changes in the same amino acid positions). Both parameters are indicative of the source-sink dynamics of adaptive evolution, in which the source is an evolutionarily primary reservoir habitat for a species and the sink is a novel and/or secondary habitat [59]. It has been hypothesized that source-sink dynamics is one of the major types of evolutionary trajectory in highly-pathogenic bacterial lineages within less pathogenic species. FimH of systemically non-invasive *Salmonella* were found to have accumulated synonymous mutations (with or without non-synonymous changes), whereas the majority of systemically invasive *Salmonella* FimH variants evolved exclusively by the accumulation of structural mutations, reflecting the recent origin of the latter. While structural mutations accompanied by synonymous mutations indicate neutral accumulation of changes, differentiation of genes exclusively through structural mutation is strong evidence of positive selection. Furthermore, the recent mutations in FimH frequently were found to be repeated hot-spot mutations, i.e. of an evolutionarily convergent nature - another strong indication that they are under positive selection and, thus, functionally adaptive.

Functional analysis of *Salmonella* FimH revealed that structural variability is associated with diverse binding properties of the adhesin, also of convergent nature, in the systemically invasive serovars. While all tested FimH variants from broad-host range serovars of *S. enterica* exhibited low binding to mannose under static conditions, FimH of host-adapted serovars had altered functional properties. FimH from systemically invasive serovars Typhi, Paratyphi C, Dublin, and some isolates of serovar Choleraesuis exhibited significantly increased binding to mannose, whereas some other FimH variants of systemically invasive serovars (Paratyphi B and Choleraesuis) did not bind mannose at all. Both the high-binding and non-binding phenotypes evolved, in part, via hot-spot mutations in different systemically invasive serovars. Of note, FimH variants of gastroenteritis–associated serovars, though differing from each other by various structural mutations, preserved low-adhesive properties suggesting the physiological importance of the low-binding FimH phenotype in the intestinal niche.

The low-binding phenotype was found to be significantly increased under shear stress, i.e., shear-enhanced in nature. This phenomenon was originally demonstrated for *E. coli* FimH and then for several other fimbrial tip-associated adhesins of enterobacteria, including *Salmonella* FimH (evolutionarily unrelated to *E. coli* FimH) [55], [60], [61], [62], [63]. The phenotype is based on an allosteric connection between the mannose-binding pocket in the lectin domain of the adhesin and the fimbria-anchoring pilin domain of FimH. When the domains separate from one another under shear-induced tensile force, the binding pocket converts from a wide-open to a tight configuration, increasing the binding affinity for mannose [64]. In contrast, the high-binding phenotype (as observed here for systemically invasive *Salmonella*) is shear-independent in nature, with already strong binding under low or no shear conditions and no enhancement of binding under shear stress. In *E. coli* FimH, the high-binding protein variants carry mutations in either lectin or pilin domains that ease the inter-domain interaction [65]. This explains why the high-binding phenotype mutations in *Salmonella* are found both in the predicted lectin (P35L, G39D and M137I) and pilin (R232W and insertion 266/267N) domains. In contrast, the non-binding phenotype mutations are found only in the lectin domain. Notably, the non-binding phenotype of *Salmonella* FimH could not be rescued under increased shear, indicating that they might directly affect the function of the mannose-binding pocket.

While the evolutionarily adaptive origin of the FimH mutations in systemically invasive *Salmonella* serovars is evident from the action of positive selection, and there are hints to their structural basis, the physiological significance of the mutations remains an open question. In *E. coli*, high-binding mutations that are selected in uropathogenic strains were shown to increase urothelial adhesion and colonization in a mouse model of infection [31]. One might speculate that in *Salmonella*, increased cell adhesiveness may also be adaptive for systemically invasive serovars. In gastroenteritis, bacterial infection remains localized to the intestine and mesenteric lymph nodes, while in systemic infections, *Salmonella* transverses the intestinal barrier and disseminates to the liver, spleen and bone marrow. A critical determinant of systemic dissemination of *Salmonella* is its ability to infect dendritic cells and other CD18-positive phagocytes [66], [67]. Recent studies of Guo at al. (2007) [39] demonstrated that *Salmonella* uptake by dendritic cells can be mediated by FimH, and the high-binding FimH variant from *S.* Typhimurium strain AJB3 was shown to be extremely efficient in mediating bacterial internalization into murine cells. Consistently, we found that FimH variants from serovars Typhi, Choleraesuis and Dublin with increased affinity towards mannose confer significantly higher adhesion to and internalization of macrophage cell line RAW 264.7 than low-binding FimH variants. Moreover, the level of RAW264.7 cell internalization mediated by these FimH variants was on average seventy five times higher than internalization of epithelial cells (Hep-2), although the level of bacterial adhesion to both of the cell types was comparable. This indicates that FimH is important factor determining bacterial entry into phagocytic cells but not into epithelial cells for which FimH-dependent internalization was only marginal. These results are consistent with previous reports demonstrating that SPI-1 encoded T3SS is the major invasive factor for epithelial cells whereas the invasion of phagocytic cells is rather SPI-1-T3SS independent [39], [68]. Although the effect of FimH-mediated entry of *Salmonella* on its intracellular survival into phagocytic cells has not been analyzed, similar studies performed in *E. coli* indicate that this route of internalization can promote bacterial survival. It was shown that FimH-dependent uptake of *E. coli* by mouse macrophages results in the formation of morphologically distinct bacteria-containing vacuoles, compared to antibody-opsonized *E. coli*, correlating with a significant increase in intracellular survival [69], [70], [71]. Thus, highly adhesive variants of FimH with increased capability to mediate internalization in phagocytes could be advantageous for the systemic dissemination of host-adapted *Salmonella* serovars.

Another important step in *Salmonella* pathogenesis is the traversing of M cells, whose apical membranes are rich in transcytotic receptor-glycoprotein 2 (GP2), a mannosylated glycoprotein that acts as a receptor for FimH [43], [44]. Although FimH variation in bacterial uptake and transcytosis by M-cells has not been studied, it is possible that allelic variations could affect bacterial delivery to deeper tissues (including Peyer's patches) and alter *Salmonella* tissue dissemination and triggering of the immune response. Of note, the mechanisms of *Salmonella* entry into M-cells appear to be important for the distribution of bacteria in tissues and activation of immune cells [72]. It has also been observed that different serovars of *Salmonella* use distinct mechanisms for M-cells transit. For example, *S.* Typhi traverses the murine epithelium via M-cells without causing M-cell destruction, followed by the rapid clearance of bacteria from Peyer patches, whereas SPI-1 T3SS-dependent invasion of M-cells by *S.* Typhimurium is accompanied by M-cell destruction, bacterial replication in Peyer's patches and robust activation of the mucosal immune response [72], [73]. Thus, in addition to the interaction with dendritic cells, allelic variations in FimH may have a significant effect on bacterial uptake by M-cells.

Somewhat unexpectedly, we discovered that some isolates of host-adapted serovars Choleraesuis and Paratyphi B carry variants of FimH that do not bind to mannose. Mannose non-binding type 1 fimbriae have been previously described for *Salmonella* in serovar Gallinarum (biovars Gallinarum and Pullorum), some isolates of Paratyphi B and Dublin, and were originally referred to as type 2 fimbriae based on the same morphology and antigenic properties as type 1 fimbriae but with an inability to cause mannose-sensitive hemagglutination [32], [74]. More recently, studies in *S.* Gallinarum revealed that non-hemagglutinating fimbriae represent type 1 fimbriae that have lost the ability to bind to mannose due to a single point mutation (T56I) in FimH [54]. However, it was also shown that although FimH of *S.* Gallinarum does not bind to murine dendritic cells or other mammalian eukaryotic cells, it does mediate efficient adhesion to chicken leukocytes in vitro and, and most recently, promote systemic dissemination of bacteria in chick model [53], [75]. These results indicate that FimH of these avian-adapted serovars of *Salmonella* can be functionally active and potentially determine *Salmonella* host-specificity. Consistent with these findings is the fact that *S.* Gallinarum FimH (Gal1 and Gal2 strains, Figure 5) has accumulated extra mutations in addition to the original mutation in *S.* Pullorum (T56I) that cause complete inactivation of FimH. The additional mutations could result in a fine-tuning of some non-mannose type of receptor of as yet unknown identity. Thus, although we did not detect adhesion of *S.* Choleraesuis or *S.* Paratyphi B non-binding FimH variants to human epithelial (Hep-2) cells and murine macrophages (RAW264.7), we cannot exclude the possibility that they are active towards a different type of eukaryotic cell or cells of different host origin.

On the other hand, it is possible that the non-binding phenotype is adaptive per se (i.e., conferring a loss-of-function) for the invasive strains. Recent whole-genome comparative analyses revealed that host-adapted) serovars of *Salmonella* have undergone extensive genome degradation [6], [7], [23], [24], with a high proportion of deleted genes or pseudogenes. Many of these pseudogenes are derived from genes of systemically non-invasive *Salmonella* that encode proteins important for intestinal colonization and intestinal persistence, including many different types of fimbriae. It has been suggested that gene silencing by pseudogene formation along with other ‘loss of function’ mutations allows host-adapted *Salmonella* to shed genes that are no longer needed in the systemic niche [7]. However, the footprint of strong positive selection for loss of function in FimH indicates that it is not removed only on a “use-it-or-lose-it” basis, but that the inactivation of FimH in invasive serovars might be adaptive for these bacteria because a functional adhesin presents a liability in the course of systemic infection. This hypothesis is in agreement with the observation that, upon oral infection of mice, a non-fimbriated *fim* mutant of *S.* Typhimurium results in significantly higher mortality than wild-type bacteria expressing type 1 fimbriae [76]. Similarly, non-type 1 fimbriated *E. coli* were selected during the course of experimentally induced bacteremia [77], [78] suggesting that attenuation of type 1 fimbrial function could be beneficial for systemic infection.

In any event, the non-binding phenotype appears to be evolutionarily linked to the highly adhesive phenotype. Indeed, in addition to both being common in systemically invasive strains, in two of three cases the high-binding variant was evolutionarily intermediate to the non-binding phenotype. On the other hand, among eight mutations leading to the high-binding phenotype of FimH in *S.* Typhi, two mutations resulted in a non-binding phenotype of FimH when tested separately, suggesting that some of the evolutionary intermediates of the human-adapted FimH could be non-binding. However, it remains to be understood how this interplay between seemingly functionally opposite phenotypes could lead to the emergence of systemically-invasive *Salmonella* from systemically non-invasive serovars. Currently, the emergence of systemically invasive non-typhoidal *Salmonella* (iNTS) strains uniquely associated with invasive diseases in humans has become a serious health problem in Africa [23]. Whole-genome sequencing of iNTS *S.* Typhimurium strain D23580 associated with these infections revealed that similarly to the host-adapted *Salmonella* this strain has undergone evolutionary changes characterized by pseudogene formation and chromosomal deletions. Analysis of *fimH* from D23580 strain and also the *Salmonella* serogroup B C-24 (Thm5) isolate from our study (with a documented clinical history of recurrent gastroenteritis/typhoid fever) showed that these strains carry the low-adhesive variant of the FimH. This indicates that these invasive disease-associated NTS strains are likely to be in earlier stages of adaptive evolution as invasive pathogens.

After orally infecting mice with recombinant *Salmonella*, we did not observe an effect of FimH mutations on bacterial burdens in the liver and spleen. However, these experiments involved only a single inoculum size and a single bacterial strain background (S. Typhimurium) constitutively expressing a plasmid copy of *fimH* used to infect a single host (BALB/c). One possible explanation of these results could be that the experiment requires most specific settings, e.g. the specific animal-host. In light of recently published findings [75], host-specificity appears to be important factor in the relevance of FimH association with *Salmonella* pathogenicity in experimental models. In the chick model, clear differences were observed in the virulence of avian adapted *Salmonella* Gallinarum with an endogenous variant of FimH and *S.* Gallinarum expressing mannose-sensitive FimH from *S.* Enteritidis. However, similar studies performed in mice showed no difference in disease for these bacteria [79]. Thus, to investigate the physiological significance of adaptive FimH variants and, in particular, their pathoadaptive role, more detailed future studies will be required.

*S. enterica* possess a wide repertoire of fimbrial and nonfimbrial adhesins that contribute to the adhesion and the pathogenicity [80], [81], [82], [83], [84], [85], with some of them differently distributed between serovars. Our studies on the mannose-specific type 1 fimbrial adhesin present in all *Salmonella* serovars demonstrate that point mutations in the FimH are acquired under positive selection and, thus, have functional consequences with an adaptive significance. However, although the FimH adhesin is the primary fimbrial subunit responsible for mannose binding, other type 1 fimbrial proteins and/or bacterial components can affect fimbriae expression and the adhesion pattern [48], [49], [86]. In addition, recently available full-genome sequences for different *Salmonella* serovars revealed the presence of potential SNP mutations and pseudogenes in *fim* structural genes and regulatory sequences raising questions about their possible influence on FimH-dependent binding [6], [24], [87]. Here, by the examination of mannose-dependent binding of wild type *Salmonella* we show that the vast majority of strains tested in our study produced type 1 fimbriae and the pattern of mannose-binding by these wild-type *Salmonella* corresponded well to the binding mediated by their FimH variants expressed in an isogenic recombinant system. These results are consistent with a previous report [32] describing type 1 fimbriae in 1444 isolates (149 serovars) of *Salmonella enterica* where most strains of most serovars were found to be fimbriated and possess mannose-dependent hemagglutinating and adhesive properties. In our study, however, some differences in fimbriation level were observed between wild-type strains, and some of the strains (*S.* Paratyphi A and *S.* Sendai) appeared to produce no fimbriae, even upon serial passage in conditions inducing type 1 fimbriae expression. Interestingly, such non-fimbriated isolates were also observed previously in Paratyphi A and Sendai serovars as well as in a portion of other serovars. This might suggest that potential loss-of-function mutations in other type 1 fimbrial genes or/and regulatory sequences could be responsible for the abrogation of type 1 fimbriae expression. However, the presence and role of these mutations/pseudogenes remain to be elucidated.

While FimH represents only one of many virulence traits, our studies clearly highlight the importance of investigating the physiological role of naturally-occurring mutations at the level of single nucleotide polymorphisms in genes shared by all *Salmonella* serovars because those mutations, in addition to horizontal gene transfer and gene loss, are likely to make a significant contribution to the adaptive evolution of *Salmonella* host adaptation and virulence.

## Materials and Methods

### Ethics statement

This study was carried out in accordance with the recommendations in the Guide for the Care and Use of Laboratory Animals of the National Institutes of Health. The protocol was reviewed and approved by the University of Washington Institutional Animal Care and Use Committee (Office of Laboratory Animal Welfare assurance number: A3464-01).

### Bacterial strains and knockouts

*Salmonella* strains used in this study are listed in Table 1. The collection included 45 isolates of *S. enterica* subspecies I (22 serovars) and 11 isolates of *S. enterica* subspecies II–VI. The strains were routinely grown overnight in LB (Luria-Bertani) broth without shaking. The non-fimbriated *fimH* mutants of *S.* Typhimurium strains SL1344H3 and LBH4 [38], [87] were used as hosts for recombinant plasmids encoding different FimH variants or fimHΔ.pISF255b plasmid with the *fimH* deletion (supplementary material Table S1). Transformed bacteria were cultured in SB (Super Broth) supplemented with 30 µg/ml chloramphenicol and 50 µg/ml kanamycin. All bacteria used in the adhesion assay were serially subcultured without shaking at 37°C for optimal expression of type 1 fimbriae. Also, for the biosafety reason, the *aroA* mutant of wild-type *S.* Typhi JSG624 (Typ4) was used in the adhesion assay. An *aroA* deletion mutant of *S.* Typhi JSG624 was constructed using λ red recombinase and primers TYP9 TYAROAP1 CTGACGTTACAACCCATCGCGCGGGTCGATGGCGCCATTAgtgtaggctggagctgcttc and TYP10- CGTACTCATCCGCGCCAGTTGTTCGAAATAATCAGGGAACcatatgaatatcctccttag as described by Datsenko and Wanner (2000). *Escherichia coli* DH5α, used for recombinant DNA manipulations, were cultured in SB broth supplemented with appropriate antibiotics as indicated.

### *fimH* sequencing and cloning

The *fimH* and three housekeeping genes (*aroC*, *hisD* and *thrA*) were PCR-amplified from various strains of *S. enterica* using genomic DNA as a template. The primers for *fimH* amplification were: fimH5′-CAGGCGATTACGATAGCC-3′ and fimH3′-ATCCACCACGTTACCGCGC-3′; and primers for housekeeping genes were as described at http://mlst.ucc.ie/mlst/dbs/Senterica. PCR products were purified after separation in 1% agarose gel on QIAquick column (Qiagen) and sequenced using BigDye Terminator v3.1 Cycle Sequencing Kit (Applied Biosystems).

For cloning, the *fimH* alleles of interest were PCR-amplified from genomic DNA using fimH-XbaI-F 5′-CTCTCTAGATGTATCCGTCCGGCGTC and fimH-SpeI-R 5′-GAGACTAGTTTAATCATAATCGACTCG-3′ primers, XbaI/SpeI digested and ligated into pISF255b [55]. The resulting plasmids carrying different *S. enterica fimH* alleles are listed in Table S1 (supplemental material).

### Site-directed mutagenesis

The different mutations were introduced into *fimH* by PCR using the Quick-Change mutagenesis kit (Stratagene). pISF255b carrying *fimH* from *S.* Typhimurium SL1344 was used as a template. The mutagenic pairs of primers are listed in Table S2 (supplemental material). Sequencing of *fimH* was performed to confirm introduced mutations.

### Phylogenetic analysis

PhyML 3.0 [88] was used to generate the maximum-likelihood based DNA phylograms of *fimH* and concatenated MLST loci, and to derive the bootstrap proportion values from 1000 replicates under GTR substitution model. The nucleotide sequences were aligned using ClustalW with default settings [89]. Zonal Phylogeny (ZP) analysis and associated statistics were performed using Zonal Phylogeny Software (ZPS) [58]. The maximum likelihood (ML) phylograms as implemented in ZPS were generated by PAUP* 4.0b using the general time reversible (GTP) substitution model with codon-position specific estimated base frequencies [90]. Sequence diversity was measured by the average pairwise diversity index (π) and the rates of nonsynonymous (dN) and synonymous (dS) mutations [91] using MEGA version 4 [92]. Analysis of statistical significance was performed using the z-test for π and dN/dS values [93]. The presence of structural hotspot mutations was determined using ZPS.

### Static adhesion assay

FimH-dependent bacterial adhesion under static condition was analyzed as described previously [94]. Briefly, immulon 4HBX 12 well strips (Thermo Electron Corp.) were coated with 20 µg/ml Man-BSA, yeast mannan, RNaseB or rabbit anti-FimH^SE^ antibody (diluted 1∶500), and quenched with 0.1% BSA in PBS. *S.* Typhimurium LBH4 expressing different FimH variants were grown overnight with 0.33 µM [methyl-^3^H]-thymidine (PerkinElmer Life Sciences, Inc.), washed with PBS and then added to each well at 100 µl volume and OD540 = 2. Plates were washed with PBS and radioactivity for each well was counted with a scintillation counter (MP Biomedicals). The number of bound bacteria was determined from calibration curves. For inhibition, bacterial binding was tested in the presence of 50 mM methyl-D-mannopyranoside (α-mm).

Some adhesion experiments were performed without ^3^H-bacteria labeling. Instead, bacteria bound to the ligands in microtiter plates were dried and then stained with 0.1% crystal violet for 10 min at room temperature. After several washes with water, 100 µl of 50% ethanol were added to each well and after dye solubilization the absorbance at a wavelength of 600 nm were measured.

### Parallel plate flow chamber experiment

Binding under flow conditions was performed using parallel plate flow chambers as described previously [55]. Briefly, 35-mm polystyrene cell culture dishes (Corning, Inc.) were coated with Man-BSA (200 µg/ml), and a parallel plate flow chamber (2.5 (long)×0.25 cm (wide)×250 µm (high), GlycoTech) was assembled on the culture dish. The entire assembly was then mounted on a Nikon TE2000-E microscope with a 10× phase-contrast objective and connected to a high resolution CCD Cascade camera (Roper Scientific, Inc.). Bacteria in 0.2% BSA/PBS were flowed into the chamber at different flow rates using a Warner Instruments syringe pump. Bacterial binding to the surface was recorded for 4 min and analyzed using MetaView video acquisition software (Universal Imaging Corp., PA).

### Cell-adhesion and invasion assays

*S.* Typhimurium SL1344H3 expressing different FimH variants were incubated with monolayers of Hep-2 or RAW 264.7 cells at a multiplicity of infection of 25∶1. Bacteria were allowed to interact with the cells for 1 h at 37°C in 5% CO_2_. The cells were then washed five times with DMEM and lysed with 1% Triton (Sigma) in PBS, or for invasion studies, incubated with DMEM containing 100 µg/ml gentamicin for 1 h at 37°C in 5% CO_2_. After antibiotic treatment the cells were washed three times with DMEM and lysed with 1% Triton. The number of CFU in each well was quantified by plating serial dilutions of cell lysates on LB plates.

### Mouse virulence assays

*S.* Typhimurium strains expressing different FimH variants from *S.* Typhimurium SL1344 (Thm1, low-binding), *S.* Typhi (Typ1, high-binding), and *S.* Choleraesuis (Chl1, inactive) were administered orally to 6–8 week-old BALB/c mice (Taconic Farms). The *fimH* genes were expressed from stable plasmid vector pRB3-273C [95]. Bacteria were grown overnight without shaking in SB broth supplemented with 30 µg/ml chloramphenicol, washed and suspended in sterile PBS (pH 7.0) to a final concentration of 10^7^ colony-forming units (CFU) per 25 µl. Food and water were withheld from the mice for 4 hours before bacteria were administered atraumatically from a pipet tip. Mice were sacrificed after 7 days for liver and spleen removal and homogenization in sterile PBS. Serial dilutions of tissue homegenates were plated onto LB agar with or without ampicillin to quantitate CFU per organ.

### List of accession/id numbers for genes mentioned in the text

EU445777- *fimH* of *S.* Typhimurium AJB3

L19338.1 - *fimH* of *S.* Typhimurium LB5010

FN424405- *fimH* of *S.* Typhimurium D23580

AY486389 - *fimH* of *S.* Gallinarum 589/02

AM933173 - *fimH* of *S.* Gallinarum 287/91

NC_012125 - *fimH* of *S.* Paratyphi C 49 [RKS 4594]

aroA gene id: 1068996

## Supporting Information

Figure S1 **Static adhesion of** ***S.*** **Typhimurium LBH4 expressing different variants of FimH to mannose-containing substrates.** Binding of ^3^H-labled bacteria to Man1 (Man-BSA) and Man5 (RNaseB) was determined as described in ‘Materials and Methods’. Data are the means ± SD of triplicates from one representative experiment of three experiments that were performed.
(TIF)

Figure S2 **Static adhesion of representative wild-type and recombinant** ***S. enterica*** **to mannose-containing substrates.** Binding of wild-type (A) and recombinant (B) *S. enterica* strains to Man1 (yeast mannan, red), Man5 (RNaseB, blue) and anti-FimH^SE^ antibody (grey). Attached bacteria were stained with crystal violet and the adhesion was quantified by measuring absorbance at 600 nm. Data are the means ± SD of triplicates from one representative experiment of three performed. The strain tags of systemically non-invasive serovars are in black and the invasive serovars in red.
(TIF)

Figure S3 **Man1/Man5 binding ratio calculated for representative wild-type and recombinant strains of** ***S. enterica.*** Data are the means ± SD of triplicates from one representative experiment of three performed. The strain tags of systemically non-invasive serovars are in black and the invasive serovars in red. ND, not determined.
(TIF)

Figure S4 **Bacterial accumulation on Man1-coated surfaces in the parallel plate flow chamber.** Bacterial binding to Man-BSA under different shear conditions was recorded for 4 min. Data are the means of two independent experiments.
(TIF)

Figure S5 **Effects of point mutations on binding phenotype of** ***S. enterica*** **FimH.** Static adhesion of ^3^H-labeled bacteria to Man1 (Man-BSA, red), Man5 (RNaseB, blue) and anti-FimH^SE^ antibody (grey). Data are the means ± SD of triplicates from one representative experiment of three performed.
(TIF)

Table S1 **List of plasmids.**
(RTF)

Table S2 **List of primers.**
(RTF)
