# Supplementary figures and images for: Evolution of Salmonella enterica Virulence via Point Mutations in the Fimbrial Adhesin
Source: PLoS Pathog. 2012 Jun 7;8(6):e1002733. doi: 10.1371/journal.ppat.1002733 (PMC3369946; doi:10.1371/journal.ppat.1002733)

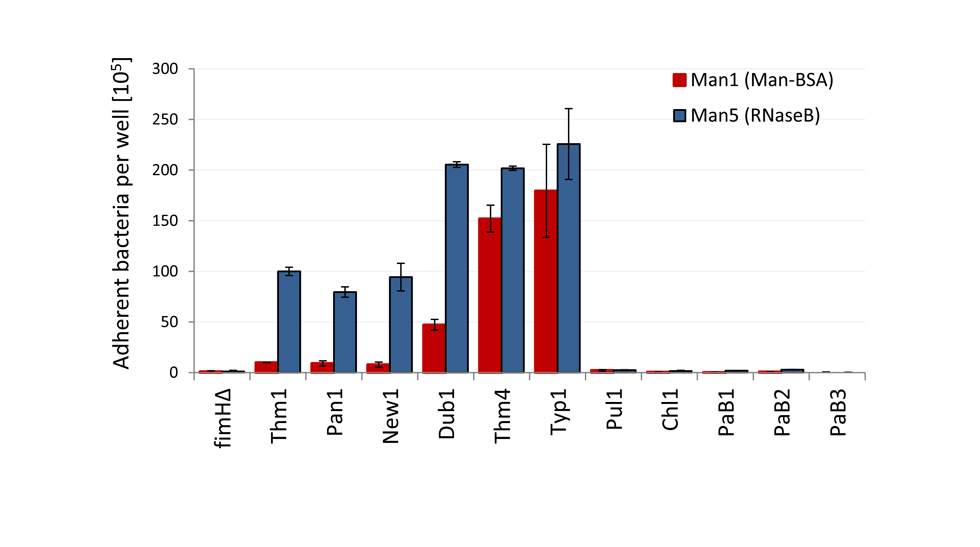

Supplement: Figure S1 — Static adhesion of S. Typhimurium LBH4 expressing different variants of FimH to mannose-containing substrates. Binding of 3H-labled bacteria to Man1 (Man-BSA) and Man5 (RNaseB) was determined as described in ‘Materials and Methods’. Data are the means ± SD of triplicates from one representative experiment of three experiments that were performed. (TIF) [file ppat.1002733.s001.tif]

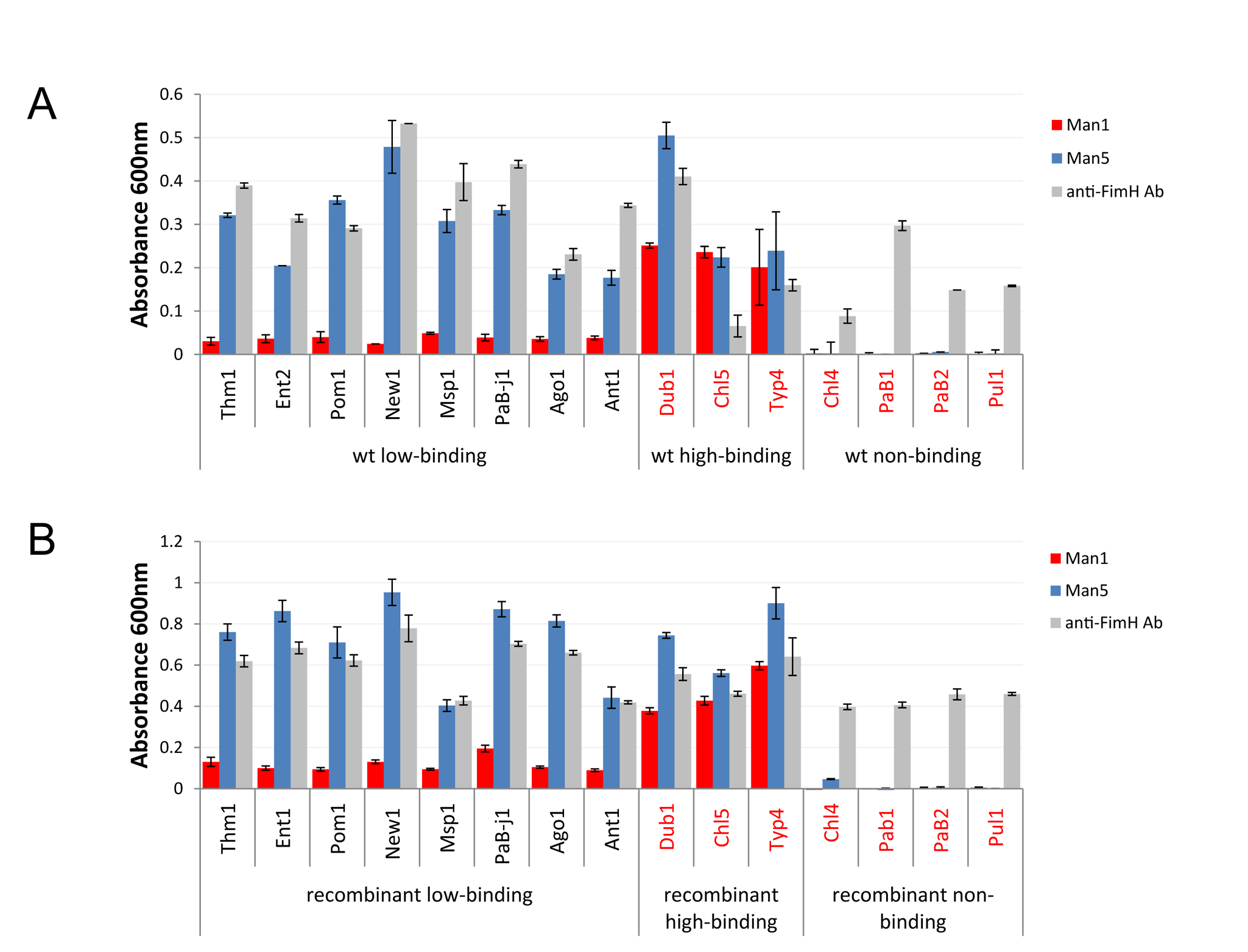

Supplement: Figure S2 — Static adhesion of representative wild-type and recombinant S. enterica to mannose-containing substrates. Binding of wild-type (A) and recombinant (B) S. enterica strains to Man1 (yeast mannan, red), Man5 (RNaseB, blue) and anti-FimHSE antibody (grey). Attached bacteria were stained with crystal violet and the adhesion was quantified by measuring absorbance at 600 nm. Data are the means ± SD of triplicates from one representative experiment of three performed. The strain tags of systemically non-invasive serovars are in black and the invasive serovars in red. (TIF) [file ppat.1002733.s002.tif]

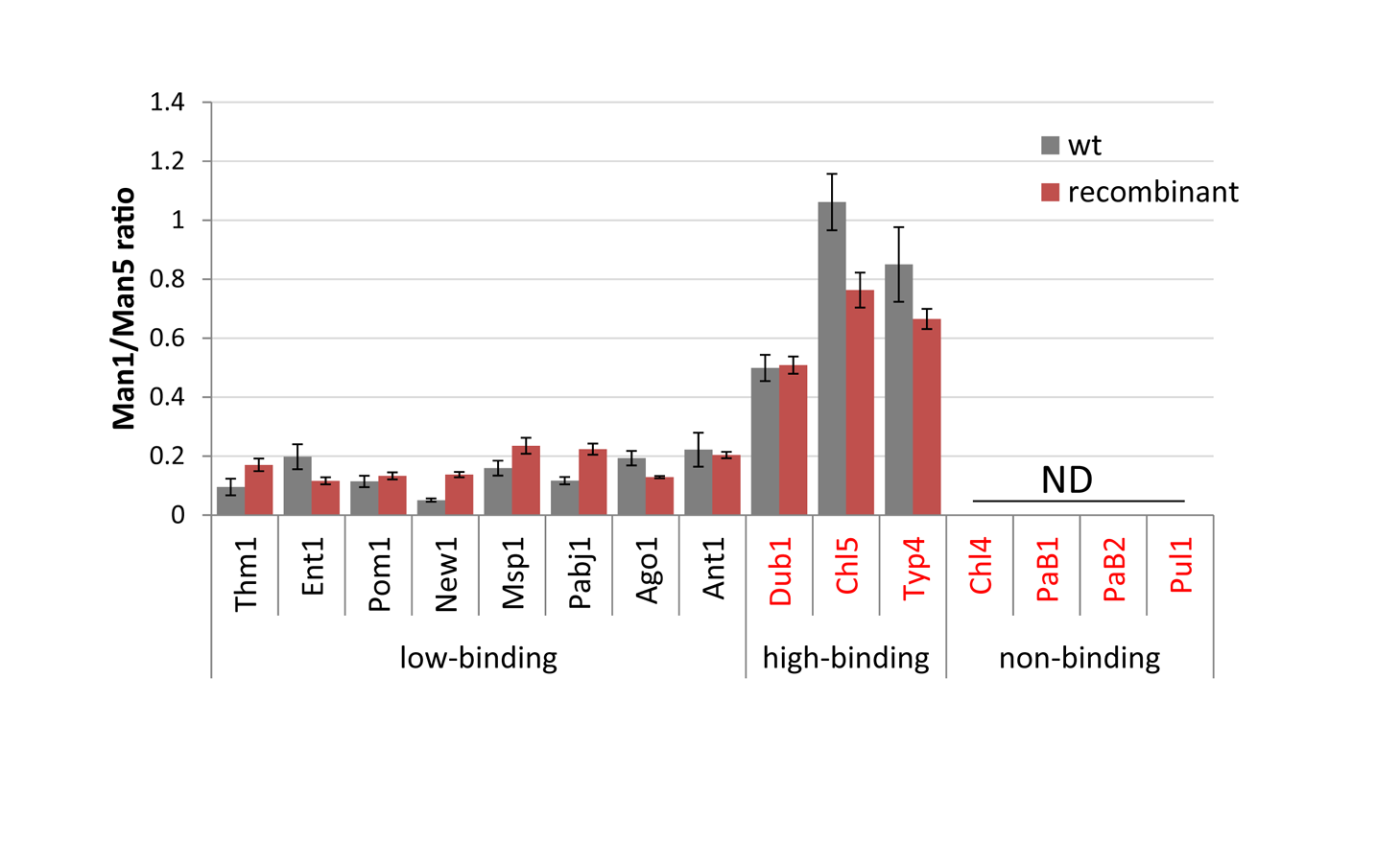

Supplement: Figure S3 — Man1/Man5 binding ratio calculated for representative wild-type and recombinant strains of S. enterica. Data are the means ± SD of triplicates from one representative experiment of three performed. The strain tags of systemically non-invasive serovars are in black and the invasive serovars in red. ND, not determined. (TIF) [file ppat.1002733.s003.tif]

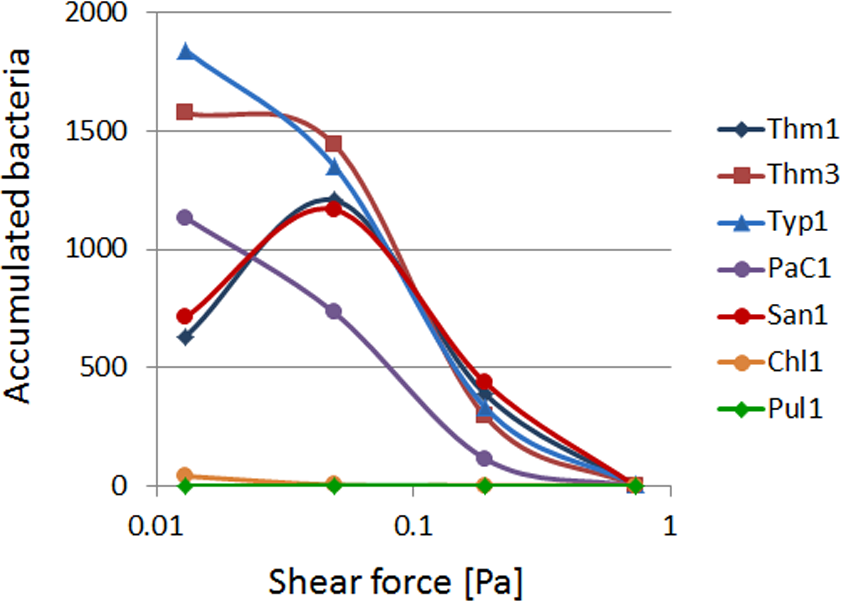

Supplement: Figure S4 — Bacterial accumulation on Man1-coated surfaces in the parallel plate flow chamber. Bacterial binding to Man-BSA under different shear conditions was recorded for 4 min. Data are the means of two independent experiments. (TIF) [file ppat.1002733.s004.tif]

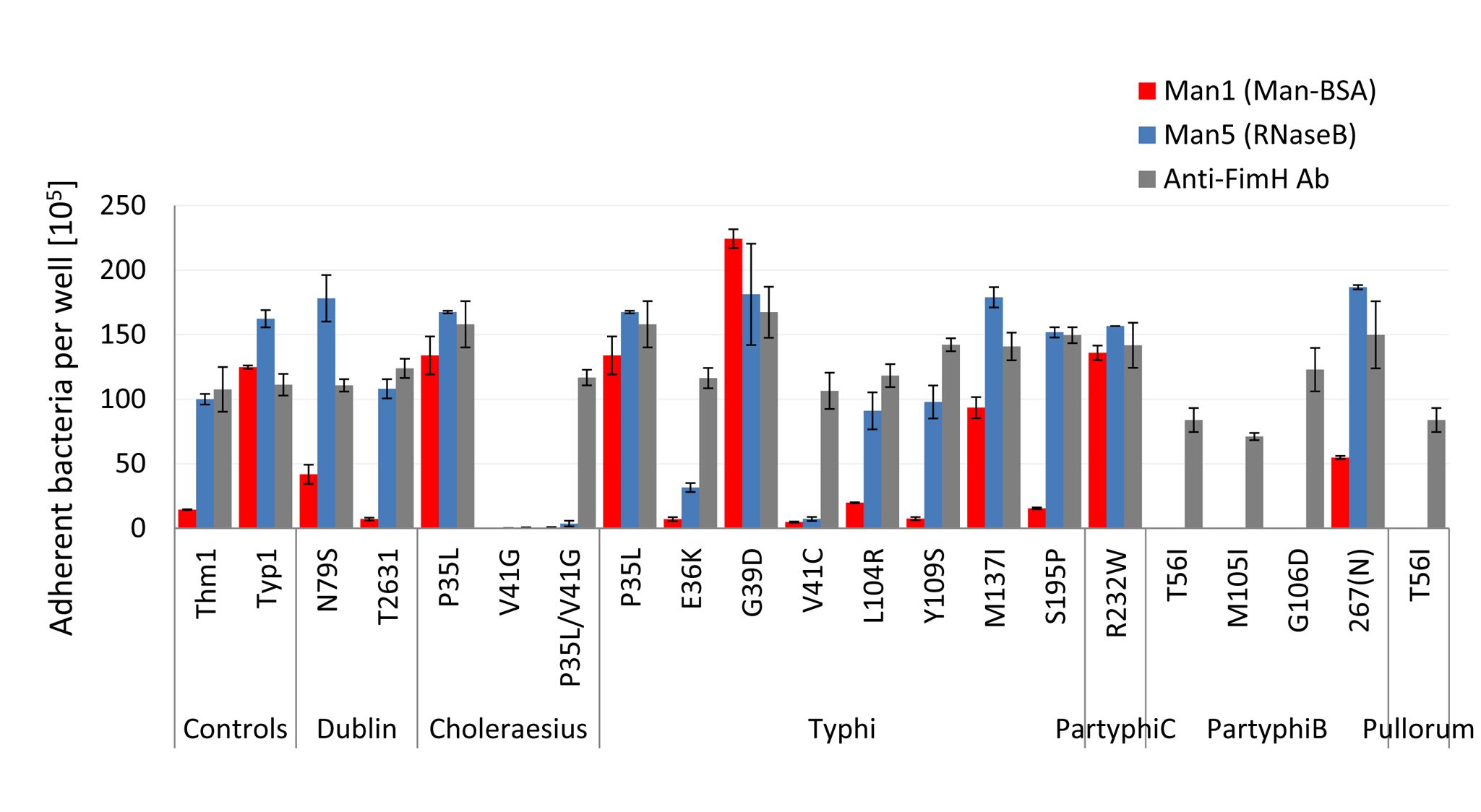

Supplement: Figure S5 — Effects of point mutations on binding phenotype of S. enterica FimH. Static adhesion of 3H-labeled bacteria to Man1 (Man-BSA, red), Man5 (RNaseB, blue) and anti-FimHSE antibody (grey). Data are the means ± SD of triplicates from one representative experiment of three performed. (TIF) [file ppat.1002733.s005.tif]
