# Supplementary material for: Evolution of Salmonella enterica Virulence via Point Mutations in the Fimbrial Adhesin
Source: PLoS Pathog. 2012 Jun 7;8(6):e1002733. doi: 10.1371/journal.ppat.1002733 (PMC3369946; doi:10.1371/journal.ppat.1002733)
Supplement: Table S1 — List of plasmids. (RTF) [file ppat.1002733.s006.rtf]

Table S1

Plasmid 	fimH origin and relevant characteristic  	Reference	
fimHPom1.pISF255b	Pom1 (S. Pomona DMSO63)	This study	
fimHAnt1.pISF255b	Ant1 (S. Anatum DMSO13)		
fimHDub1.pISF255b	Dub1 (S. Dublin DMSO30)		
fimHMis1.pISF255b	Mis1 (S. Mississippi DMSO49)		
fimHAgo1.pISF255b	Ago1 (S. Agona DMSO09)		
fimHNew1.pISF255b	New1 (S. Newport DMSO55)		
fimHPul1.pISF255b	Pul1 (S. Pullorum SL297)		
fimHPab1.pISF255b	PaB1 (S. Paratyphi B S66)		
fimHPab2.pISF255b	PaB2 (S. Paratyphi B S957)		
fimHPab3.pISF255b	PaB3 (S. Paratyphi B 2421)		
fimHPaB-j1.pISF255b	PaB-j1 (S. Paratyphi B var java DMSO45)		
fimHEnt1.pISF255b	Ent1 (S. Enteritidis 11122-1)		
fimHChl1.pISF255b	Chl1 (S. Choleraesuis ÷3246)		
fimHChl5.pISF255b	Chl5 (S. Choleraesuis 1656/04)		
fimHTyp1.pISF255b	Typ1 (S. Typhi RKS 3333)		
fimHPaA1.pISF255b	PaA1 (S. Paratyphi A RKS 4993)		
fimHInd1.pISF255b	Ind1 (S. Indiana RKS 4250)		
fimHMun1.pISF255b	Mun1 (S. Muenchen  RKS 3121)		
fimHWie1.pISF255b	Wie1 (S. Wien RKS 4000)		
fimHPoa1.pISF255b	Poa1 (S. Poona MI14a)		
fimHPan1.pISF255b	Pan1 (S. Panama LI03e)		
fimHSan1.pISF255b	San1 (S. Sandiego MI08d)		
			
fimHThm1.pISF255b	Thm1 (S. Typhimurium SL1344)	Kisiela et al., 2011	
fimHThm3.pISF255b	Thm3 (S. Typhimurium AJB3)		
fimHThm4.pISF255b	Thm4 (S. Typhimurium LB5010)		
fimHÄ.pISF255b	fimH deletion of pISF255b		
pISF255b	modified pISF255 plasmid with SpeI restriction site introduced between fimH and fim FZ  		
			
pISF255	pACYC184 carrying fim HFZ  segment from S. Typhimurium SL1344 	Boddicker et al., 1998	
