# Supplementary material for: Evolution of Salmonella enterica Virulence via Point Mutations in the Fimbrial Adhesin
Source: PLoS Pathog. 2012 Jun 7;8(6):e1002733. doi: 10.1371/journal.ppat.1002733 (PMC3369946; doi:10.1371/journal.ppat.1002733)
Supplement: Table S2 — List of primers. (RTF) [file ppat.1002733.s007.rtf]

Table S2

Primer Name	Sequence 5'to 3'	
N79S-F	TACCTCAAGTTGAGTGACTACCTTCTG	
N79S-R	CAGAAGGTAGTCACTCAACTTGAGGTA	
T263I-F	GTACGCCGCTTATACCCAATAATTTG	
T263I-R	CAAATTATTGGGTATAAGCGGCGTAC	
M105I-F	CCGTAACTATATTCTCATCGGCGTCGACT	
M105I-R	AGTCGACGCCGATGAGAATATAGTTACGG	
G106D-F	CTATATTCTCATGGACGTCGACTATAAC	
G106D-R	GTTATAGTCGACGTCCATGAGAATATAG	
267(N)-F	CTTACACCCAATAATAACTTGTCGAGTAAAATTCC	
267(N)-R	GGAATTTTACTCGACAAGTTATTATTGGGTGTAAG	
R232W-F	ATTTATCTATGTGGCTTGAAGCCGA	
R232W-R	TCGGCTTCAAGCCACATAGATAAAT	
V41G-F	ATCAGGTTGGGGCGGCGTAAACGCG	
V41G-R	CGCGTTTACGCCGCCCCAACCTGAT	
P35L-F	GTGGTGACGCTGCTGGAAAAATCAGGTTGG	
P35L-R	CCAACCTGATTTTTCCAGCAGCGTCACCAC	
V41C-F	ATCAGGTTGGTGCGGCGTAAACGCG	
V41C-R	CGCGTTTACGCCGCACCAACCTGAT	
L104R-F	CCGTAACTATATTCGCATGGGCGTC	
L104R-R	GACGCCCATGCGAATATAGTTACGG	
Y109S-F	TGGGCGTCGACTCTAACGTGTCGCAG	
Y109S-R	CTGCGACACGTTAGAGTCGACGCCCA	
E36K-F	GGTGACGCTGCCGAAAAAATCAGGTTGG	
E36K-R	CCAACCTGATTTTTTCGGCAGCGTCACC	
M137I-F	CCTTTTATTAATATAGTGACGATCCC	
M137I-R	GGGATCGTCACTATATTAATAAAAGG	
S195P-F	GATATCGGCGCGCCGTTATTTAGTCAG	
S195P-R	CTGACTAAATAACGGCGCGCCGATATC	
G39D-F	CCGGAAAAATCAGATTGGGTCGGC	
G39D-R	GCCGACCCAATCTGATTTTTCCGG	
T56I-F	CAACGGTGAATTATATCTACCGAAGCTATGTATC	
T56I-R	CATACATAGCTTCGGTAGATATAATTCACCGTTG	
